# Improvement of glycemic control and reduction of major cardiovascular events in 18 cardiovascular outcome trials: an updated meta-regression

**DOI:** 10.1186/s12933-021-01401-8

**Published:** 2021-10-18

**Authors:** Maria Ida Maiorino, Miriam Longo, Lorenzo Scappaticcio, Giuseppe Bellastella, Paolo Chiodini, Katherine Esposito, Dario Giugliano

**Affiliations:** 1 Diabetes Unit, Department of Advanced Medical and Surgical Sciences, University of Campania Luigi Vanvitelli, Naples, Italy; 2 Division of Endocrinology and Metabolic Diseases, Department of Advanced Medical and Surgical Sciences, University of Campania Luigi Vanvitelli, Naples, Italy; 3 Medical Statistics Unit, University of Campania Luigi Vanvitelli, Naples, Italy

**Keywords:** Cardiovascular outcome trials, Type 2 diabetes, DPP-4i, GLP-1RA, SGLT-2i, Cardiorenal outcomes, Meta-regression, MACE, Glycemic control

## Abstract

**Background:**

Besides providing reassurance about cardiovascular (CV) safety of newer diabetes drugs, cardiovascular outcome trials (CVOTs) have also shown encouraging benefits on some CV endpoints. The contribution of the better glycemic control in the reduction of major cardiovascular events (MACE) remains an open question. The aim of this study is to evaluate the associations between the reduction of HbA1c and risk of MACE, MACE components, hospitalization for heart failure (HF) and all-cause death in CVOTs.

**Methods:**

An electronic search up to July 2021 was conducted to determine eligible trials. Systematic review identified eighteen CVOTs reporting prespecified CV outcomes. Pooled summary estimates and 95% confidence intervals (CI) were calculated according to the random effects model using the Paule-Mandel method; restricted maximum likelihood estimators were used to estimate model parameters in the metaregression.

**Results:**

The eighteen CVOTs evaluated 161,156 patients and included four trials with dipeptidyl-peptidase-4 inhibitors (DPP-4i), eight trials with glucagon-like peptide-1 receptor agonists (GLP-1RA) and six trials with sodium-glucose cotransporter-2 inhibitors (SGLT-2i). Random-effects model meta-analysis showed an association between treatment and risk of MACE (hazard ratio [HR] 0.90; 95% CI 0.86, 0.94, P < 0.001), with significant heterogeneity between studies (I^2^ = 45.2%, Q statistic P = 0.040). In meta-regression, there was an association between the reduction in HbA1c at the end of the trial and the HR reduction for MACE (beta =  − 0.298, P = 0.007), with significant heterogeneity (I^2^ = 40%, Q statistic P = 0.04); this association was totally driven by the risk reduction of non-fatal stroke, which explained 100% of between-study variance (beta =  − 0.531, R^2^ = 100%), without heterogeneity (I^2^ = 24%, Q statistic P = 0.206). There was no association between the reduction in HbA1c and the HR for heart failure or all-cause death.

**Conclusions:**

The reduction of HbA1c in eighteen CVOTs was significantly associated with reduction of non-fatal stroke, explaining all (R^2^ = 100%) of the between-study variance. While the contribution of glucose lowering in some CV benefits of newer agents does not influence their indications for the patient with type 2 diabetes, it may hopefully facilitate their use.

**Supplementary Information:**

The online version contains supplementary material available at 10.1186/s12933-021-01401-8.

## Introduction

All the dedicated cardiovascular outcome trials (CVOTs), designed to ensure cardiovascular (CV) safety of the newer glucose-lowering drugs, have provided reassurance about their overall CV safety, and have also shown some encouraging benefits on CV endpoints. Meta-analyses of CVOTs suggest that glucagon-like peptide-1 receptor agonists (GLP-1RA) and sodium-glucose cotrasporter-2 inhibitors (SGLT-2i) reduce the risk of major adverse cardiovascular events (MACE) to a comparable degree [1–5]. Accordingly, in patients with type 2 diabetes and established CV disease or multiple CV risk factors, GLP-1RA or SGLT-2i with demonstrated CV benefits are recommended to reduce the risk of MACE [6, 7].

All the CVOTs were designed to reach glycemic equipoise between groups, for minimizing the confounding effect of different glycemic control, and for not exposing participants to the increased risk of microvascular complications, a likely consequence of the suboptimal glycemic control [8]*.* At the end of CVOTs, however, subjects in the placebo groups had worse glycemic control as compared with those in the treatment groups. In some CVOTs, the imbalance of glycemic control between groups was equal to or greater than that obtained in the UKPDS 34 study [9], the first trial of intensive glycemic control to explore the supposed beneficial effects of near normal hyperglycemia. This imbalance may have masked potential benefits of the glycemic control per se on the CV endpoint. As stated by FDA [10], whether these differences in glycemic control could have contributed directly or indirectly to differences in observed outcomes (i.e., the outcome MACE) is unknown.

The contribution of the better glycemic control in the mediation of the parallel reduction of MACE in CVOTs remains an open question. New diabetes drugs include glucose-lowering agents which were the last to be introduced in the market for the use in people with type 2 diabetes, and specifically both incretins [GLP-1RA and dipeptidyl-peptidase 4 inhibitors—(DPP-4i)] and SGLT-2i. The low penetration of these newer drugs in the U.S. market (7.1% for both GLP-1RA and SGLT-2i in the last NHANES wave) [11] could also have been a consequence of the lack of enthusiasm of diabetes specialists who are still reluctant to admit that antihyperglycemic drugs may only work independently of blood glucose regulation. A previous meta-analysis with meta-regression of the 15 CVOTs published until 2020 [4] reported a robust association between the reduction in achieved HbA1c at the end of the trial and the reduction of the risk for MACE, which was driven by the risk reduction of non-fatal stroke only, and apparently restricted to the class of GLP-1RA. The recent availability of three new CVOTs, one involving the SGLT-2i ertuglflozin (VERTIS-CV) [12], one involving the SGLT-1/2 inhibitor sotagliflozin (SCORED) [13], and one involving the long-acting GLP-1RA efpeglenatide (AMPLITUDE-O) [14], offer the opportunity to further disclose whether the improved glycemic control associated with the use of newer glucose-lowering drugs play a role in ameliorating the cardiovascular outlook of people with type 2 diabetes. Therefore, the purpose of this study was to use meta-analysis and meta-regression to investigate the association between improvement of glycemic control, as assessed by reduction of HbA1c during active treatment, and risk of MACE, MACE components, hospitalization for heart failure (HF) and all-cause death in all CVOTs published to date.

## Research design and methods

### Search strategy and eligibility criteria

We conducted this systematic review and meta-analysis based on Preferred Reporting Items for Systematic Reviews and Meta-analyses (PRISMA) guidelines [15]. The PRISMA checklist and the protocol are provided in Additional file 1. Neither ethics approval nor patient consent was required for this analysis. The protocol has not been registered on any platform.

Databases searched included PubMed, EMBASE, the Cochrane Central Register of Controlled Trials, the Cochrane Database of Systematic Reviews, and ClinicalTrials.gov (http://www.clinicaltrials.gov). The last search was performed on July 10, 2021. The search terms used were ‘dipeptidyl-peptidase inhibitor (DPP-4i)’, ‘saxagliptin’, ‘alogliptin’, ‘sitagliptin’, ‘linagliptin’, ‘glucagon-like peptide-1 receptor agonist’, ‘exenatide’, ‘lixisenatide’, ‘liraglutide’, ‘semaglutide’, ‘dulaglutide’, ‘albiglutide’, ‘efpeglenatide’, ‘sodium-glucose co-transporter-2 inhibitor’, ‘empagliflozin’, ‘canagliflozin’, ‘dapagliflozin’, ‘ertugliflozin’, ‘sotagliflozin’, ‘major cardiovascular events’, ‘MACE’, ‘myocardial infarction’, ‘stroke’, ‘hospitalization for heart failure’, ‘cardiovascular death’ and ‘all-cause death’. The search was filtered to include only randomized controlled trials (RCTs) or meta-analyses involving humans. Reference lists of prior reviews and meta-analyses were also manually searched to capture other relevant RCTs.

We included trials if they were RCTs performed in adults with type 2 diabetes, compared add-on therapy with any DPP-4i, GLP-1RA or SGLT-2i against placebo, and included in the outcome (either primary or secondary) MACE, as well as other outcomes required by regulatory agencies for CV safety studies in diabetes. We excluded trials if they were completed before the FDA guidance of 2008 [16]. Results reported in trial publications were used as the primary source of information. Available additional sources, including, but not limited to, the FDA, European Medicines Agency, and pharmaceutical company websites, were searched to capture any relevant additional data.

### Data extraction and quality assessment

Two investigators (M.I.M. and D.G.) used a standardized tool (provided in Additional file 1) to independently abstract all data. The relevance of studies was assessed with a hierarchical approach based on title, abstract and the full manuscript. After the initial screening of titles and abstracts, the studies included were compared, and any disagreements were resolved by consensus. We evaluated the risk of bias of the included RCTs according to the Cochrane Collaboration's tool for assessing the risk of bias [17].

### Outcomes

The primary outcomes for this meta-analysis were to disclose associations between reductions in HbA1c and risk of MACE with meta-regression, and to assess the effect of DPP-4i, GLP-1RAs and SGLT-2i on the risk of MACE. Additional preplanned analyses were conducted on the incidence of MACE components (CV death, non-fatal myocardial infarction [MI], non-fatal stroke), hospitalization for HF and all-cause death, as well as on associations between reductions in HbA1c and their risks with meta-regression.

### Data synthesis and analysis

Data were analyzed using Stata version 16.0 (Stata Corporation, College Station, TX, USA). Hazard ratios (HRs) and 95% confidence intervals (95% CI) were collected for CV efficacy outcomes. Heterogeneity between studies was assessed by using the Q statistic and I^2^: I^2^ < 25% was considered as low in heterogeneity, I^2^ > 75% as high in heterogeneity, and a Q statistic P-value of < 0.10 was considered significant. Pooled summary estimates and 95% CI for CV efficacy outcomes were calculated according to a random-effects model using the Paule-Mandel method [18]. Publication bias was assessed visually with funnel plots and with the Egger test [19]. The trim-and-fill method [20] was used to estimate the effect of publication bias, if any.

We performed a meta-regression analysis of the CVOTs to describe the relationship between the differences in achieved HbA1c at the end of CVOTs and the corresponding HR for MACE, as well as other CV outcomes. The meta-regression relates the treatment effect to study-level covariates while assuming additivity of within-study and between-studies components of variance. Restricted maximum likelihood estimators were used to estimate model variables [21]. A permutation test (using 1000 reallocations) was used for assessing the true statistical significance of an observed meta-regression finding [22]. Beta on log HR for an absolute change of 1% in HbA1c was also reported.

## Results

### Individual Trial Characteristics

Of 242 articles screened for eligibility, 18 trials [12–14, 23–37] were eligible and included in the meta-analysis (Additional file 1: Figure S1). There were four trials with DPP-4i, eight trials with GLP-1RA and six trials with SGLT-2i. Their characteristics are summarized in Table 1. The participants were all patients with type 2 diabetes (aged > 18 years old). All trials were multinational and sponsored by industry. The trials have been published during 2013–2021, with two studies published in 2021. All trials were of parallel-group, double-blind design, and their mean duration ranged from 1.5 to 5.4 years. The baseline HbA1c level ranged from 7.3% (56 mmol/mol) to 8.9% (74 mmol/mol) but was almost identical between groups (drug vs. placebo) within the same trial. The populations studied ranged in size from 3183 (PIONEER 6) to 17,160 (DECLARE) and were of similar age (range: 60–69 years).

**Table 1:** Key baseline characteristics from each CVOT

Trial/year of publication	Study drug/follow-up (y)	Participants (n)	Mean age years	Baseline HbA1c	Prior CVD (%)	Study funder
DPP-4i						
SAVOR-TIMI 53	Saxagliptin	16,492	65.0	8.0%	78.6	AstraZeneca
2013	2.1			64 mmol		Bristol-Myers
EXAMINE	Alogliptin	5380	61.0	8.0%	100	Takeda
2013	1.5			64 mmol		
TECOS	Sitagliptin	14,6671	66.0	7.3%	74	Merck Sharp &
2015	2.8			56 mmol		Dohme
CARMELINA	Linagliptin	6979	65.9	7.9%	100	Boehringer
2018	2.2			63 mmol		Ingelheim
GLP-1RA						
ELIXA	Lixisenatide	6068	60	7.7%	100	Sanofi
2015	2.1			61 mmol		
LEADER	Liraglutide	9340	64.3	8.7%	72.4	Novo-Nordisk
2016	3.8			72 mmol		
SUSTAIN-6	Semaglutide	3297	64.6	8.7%	83.0	Novo-Nordisk
2016	3.1			72 mmol		
EXSCEL	Exenatide OW	14,752	62.0	8.0%	73.1	Amylin Pharma-
2017	3.2			64 mmol		ceuticals
HARMONY	Albiglutide	9463	64.0	8.7%	100	GlaxoSmithKline
2018	1.6			72 mmol		
REWIND	Dulaglutide	9901	66.2	7.2%	31.4	Boehringer/Lilly
2019	5.4			55 mmol		
PIONEER 6	Semaglutide O	3183	66.0	8.2%	84.7	Novo-Nordisk
2019	1.3			66 mmol		
AMPLITUDE-0	Efpeglenatide	4076	64.5	8.9%	89.6	Sanofi
2021	1.8			74 mmol		
SGLT-2i						
EMPA-REG	Empagliflozin	7021	63.2	8.1%	100	Boehringer/Lilly
2015	3.1			65 mmol		
CANVAS	Canagliflozin	10,142	63.2	8.2%	72.2	Janssen
2017	2.4			66 mmol		
DECLARE	Dapagliflozin	17,160	63.8	8.3%	40.6	AstraZeneca
2017	4.2			67 mmol		
CREDENCE	Canagliflozin	4401	63.0	8.3%	50.4	Janssen
2019	2.6			67 mmol		
VERTIS-CV	Ertugliflozin	8246	64.4	8.2%	100	Merck Sharp &
2020	3.0			66 mmol		Dohme
SCORED	Sotagliflozin	10,584	69.0	8.3%	48.6	Sanofi/Lexicon
2021	1.4			67 mmol		Pharmaceuticals

*OW* once weekly, *O* oral

The 18 CVOTs evaluated 161,156 patients and the following classes of medications: DPP-4i (saxagliptin, alogliptin, sitagliptin, linagliptin) in 43,522 participants; GLP-1RA (lixisenatide, liraglutide, semaglutide, exenatide once-weekly, albiglutide, dulaglutide, oral semaglutide, and efpeglenatide) in 60,080 participants; and SGLT-2is (empagliflozin, canagliflozin, dapagliflozin and sotagliflozin) in 57,554 participants. There was no major risk of bias (Cochrane tool) in any study (Additional file 1: Figure S2). In all trials, MACE was the primary outcome, except CREDENCE and SCORED, in which MACE was a secondary outcome.

### MACE

**Fig. 1:**
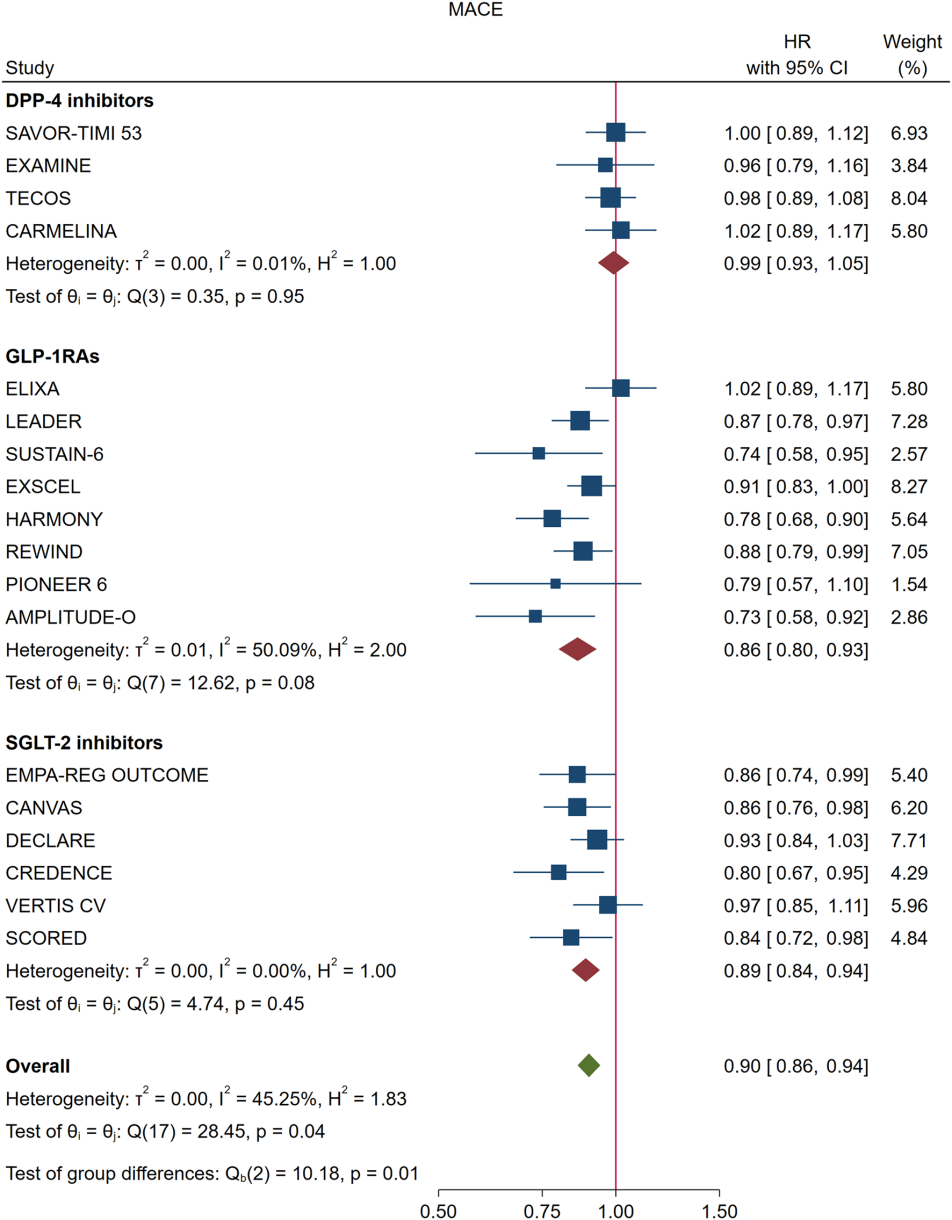
Forest plot of risk (HR, hazard ratio) of MACE (major cardiovascular events) with the use of DPP-4i, GLP-1RA or SGLT-2i, as compared with placebo, in patients with type 2 diabetes participating in 18 CVOTs (cardiovascular outcome trials)

**Fig. 2:**
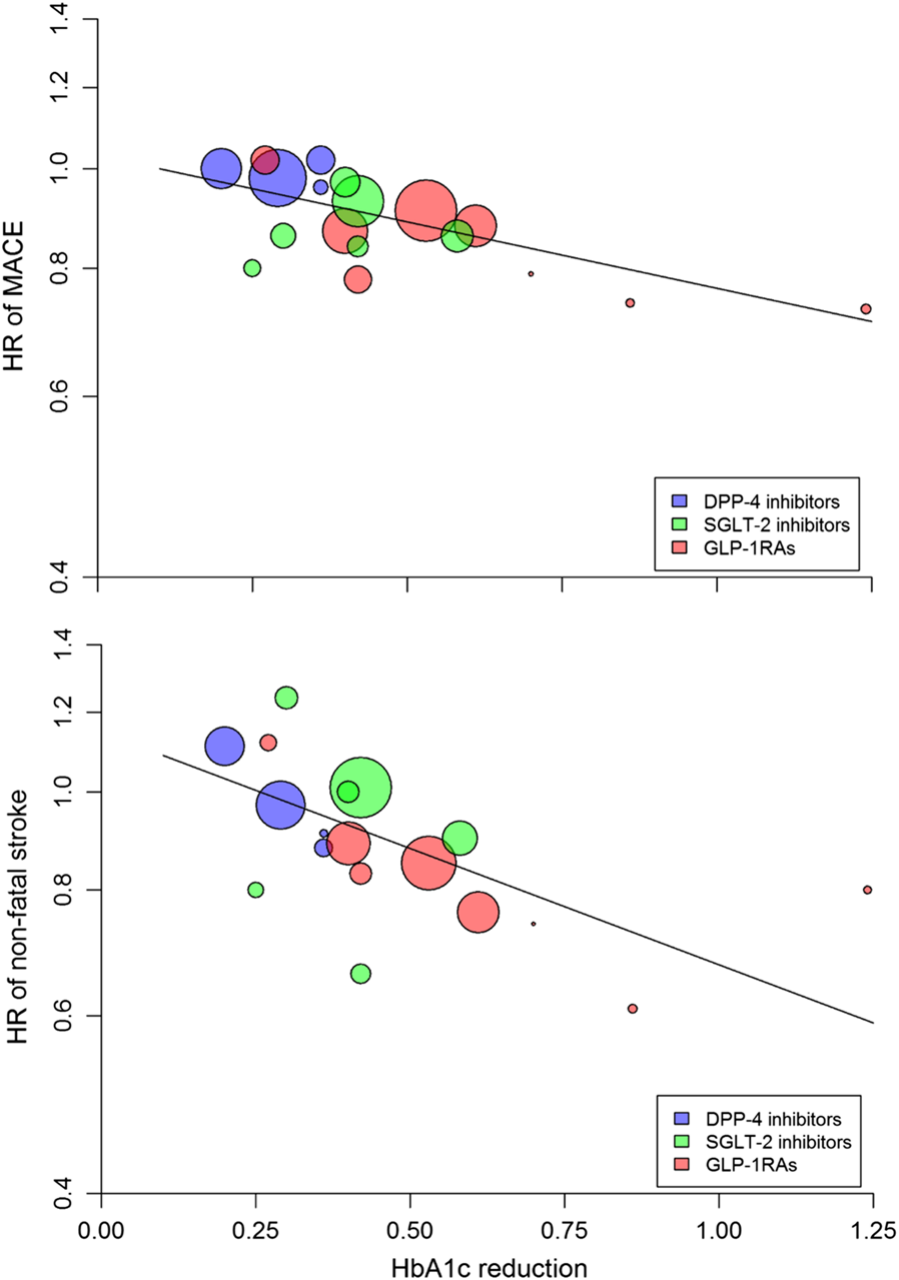
Meta-regression analysis between the differences (intervention minus placebo) in achieved HbA1c at the end of CVOTs and the corresponding hazard ratio (HR) for MACE (top) or non-fatal stroke (bottom) in patients with type 2 diabetes participating in 18 CVOTs

In the pooled analysis of 18 trials, the risk of MACE was significantly reduced by 10% (HR = 0.90, 0.86–0.94, P < 0.001) compared with placebo, with significant heterogeneity between trials (I^2^ = 45.2%, P = 0.040) (Fig. 1, Additional file 1: Table S1) and evidence of publication bias (Egger test, P = 0.016). The trim-and-fill method indicated that this publication bias did not change the statistical significance of the estimate (HR 0.91, 95% CI 0.87–0.96). Meta-regression showed an association (Fig. 2) between the reduction in achieved HbA1c at the end of the trial and the HR reduction for MACE (beta =  − 0.298, P = 0.007), explaining almost all (97%) of the between-study variance (Table 2). For every 1% (10.93 mmol/mol) greater average reduction in HbA1c, the risk of MACE decreased by 26%. Compared with placebo, DPP-4i showed a neutral effect on MACE, while the use of both GLP-1RA and SGLT-2i was associated with significant reductions in MACE (14% and 11%, respectively), with non-significant heterogeneity for SGLT-2i (Fig. 1, Additional file 1: Table S1). The trials that showed a significant benefit on MACE were LEADER, SUSTAIN 6, HARMONY, REWIND and AMPLITUDE-O for GLP-1RA, and EMPA-REG OUTCOME, CANVAS, CREDENCE, and SCORED for SGLT-2i (Fig. 1).

**Table 2:** Values of heterogeneity and Beta on log HR in the meta-regression

	I^2^	P-value of heterogeneity	Beta on log (HR)	P-value	R^2^
MACE	40%	0.040	− 0.298	0.007	97%
CV death	39%	0.047	− 0.176	0.311	4%
Non-fatal MI	30%	0.108	− 0.181	0.256	3%
Non-fatal stroke	21%	0.206	− 0.531	0.008	100%
Heart failure	70%	< 0.001	− 0.186	0.474	0%
All-cause death	48%	0.012	− 0.196	0.192	24%

### Cardiovascular death

**Fig. 3:**
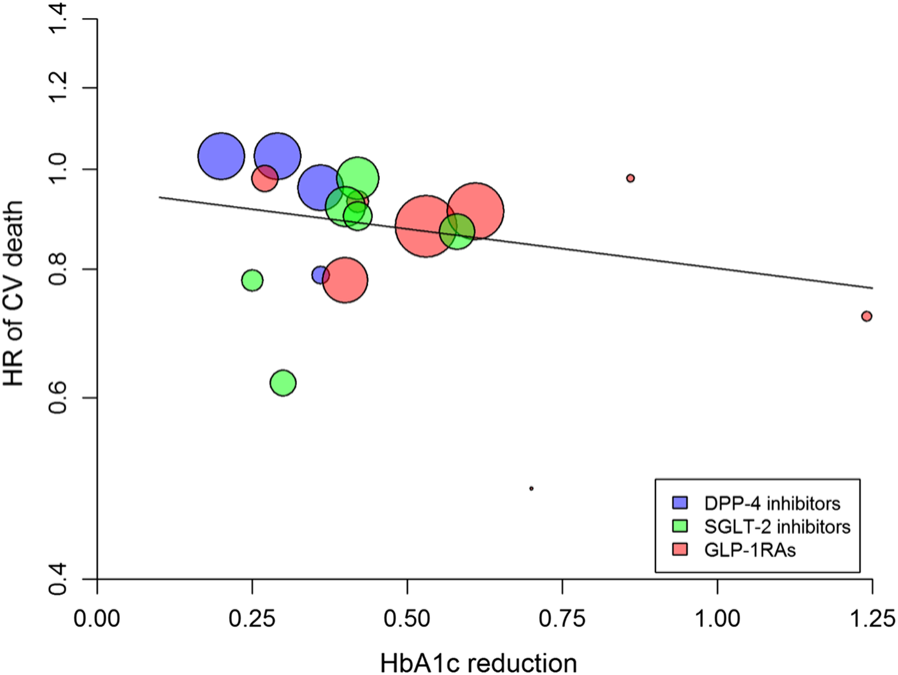
Meta-regression analysis between the differences in achieved HbA1c at the end of cardiovascular outcome trials (CVOTs) and the corresponding hazard ratio (HR) for CV death in patients with type 2 diabetes participating in 18 CVOTs

There was a significant (P < 0.001) 12% risk reduction in CV death associated with the use of newer drugs compared with placebo, with significant heterogeneity (I^2^ = 39%, P = 0.047) (Table 2) and evidence of publication bias (Egger test, P = 0.028). However, the trim-and-fill method indicated that this publication bias did not impact the estimate. The association between the reduction in achieved HbA1c and CV death was not significant (beta =  − 0.176, P = 0.311, variance explained = 4%) (Fig. 3, Table 2). The use of both GLP-1RA and SGLT-2i was associated with a significant 13% and 16% risk reduction in CV death, respectively, with non-significant heterogeneity for GLP-1RA (Additional file 1: Table S1). The trials that showed a clear significant benefit on CV death were LEADER and PIONEER 6 for GLP-1RA, and EMPA-REG OUTCOME for SGLT-2i.

### Non-fatal myocardial infarction

**Fig. 4:**
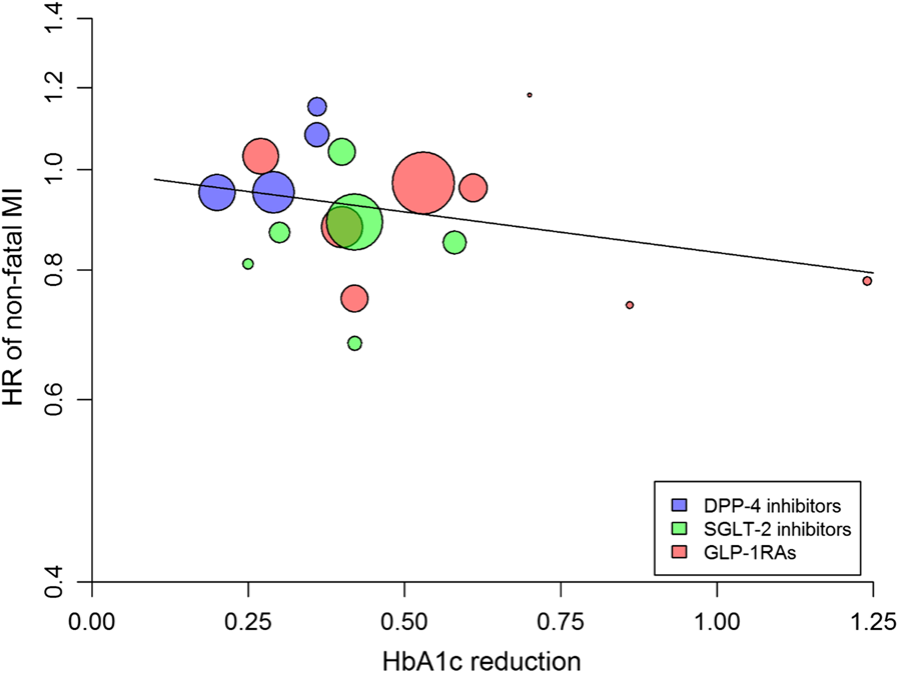
Meta-regression analysis between the differences in achieved HbA1c at the end of cardiovascular outcome trials (CVOTs) and the corresponding hazard ratio (HR) for non-fatal MI in patients with type 2 diabetes participating in 18 CVOTs

Overall, there was a significant (P = 0.007) 8% risk reduction of non-fatal MI associated with the use of newer drugs compared with placebo, with non-significant heterogeneity (I^2^ = 36.5%, P = 0.108) (Additional file 1: Table S1) and no evidence of publication bias (Egger test, P = 0.364). The association between the reduction in achieved HbA1c and nonfatal MI was not significant (beta =  − 0.181, P = 0.256, variance explained = 3%) (Fig. 4, Table 2). The use of both GLP-1RA and SGLT-2i was associated with a significant 9% and 13% risk reduction in CV death, respectively, with non-significant heterogeneity for both (Additional file 1: Table S1). No single trial in any class produced a significant benefit on non-fatal MI, except for albiglutide (HARMONY trial) and sotagliflozin (SCORED trial).

### Non-fatal stroke

There was a significant (P = 0.012) 9% risk reduction of non-fatal stroke associated with the use of newer drugs, which was largely driven by the 16% reduction associated with the use of GLP-1RA (Additional file 1: Table S1), with no significant heterogeneity (I^2^ = 21.3%, P = 0.206) and no evidence of publication bias (Egger test, P = 0.233). The association between the reduction in achieved HbA1c and non-fatal stroke was highly significant (beta =  − 0.531, P = 0.008, variance explained = 100%) (Table 2) and accounted for almost all the association between MACE and HbA1c reduction. For every 1% (10.93 mmol/mol) greater average reduction in HbA1c, the risk of non-fatal stroke decreased by 41%. The trials that showed a clear significant benefit on non-fatal stroke were SUSTAIN-6 and REWIND for GLP-1RA and SCORED for SGLT-2i.

### Hospitalization for HF

**Fig. 5:**
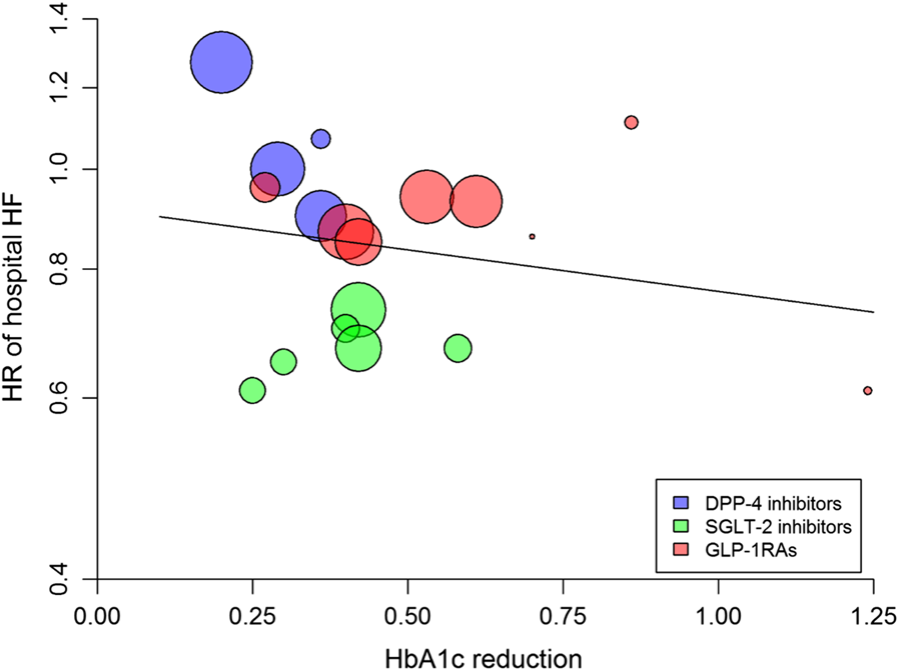
Meta-regression analysis between the differences in achieved HbA1c at the end of cardiovascular outcome trials (CVOTs) and the corresponding hazard ratio (HR) for hospitalization for heart failure (HF) in patients with type 2 diabetes participating in 18 CVOTs

In pooled analysis of the 18 trials, the risk of hospitalization for HF showed a significant (P < 0.001) 16% reduction with the newer antihyperglycemic drugs, with significant heterogeneity between trials (I^2^ = 69.2%, P < 0.001) (Additional file 1: Table S1), and no evidence of publication bias (Egger test, P = 0.413). There was no association between the reduction in achieved HbA1c and the HR for HF (beta =  − 0.186, P = 0.474, variance explained = 0%) (Fig. 5, Table 2). The use of both GLP-1RA and SGLT-2 was associated with significant reductions of the HF risk (10% and 32%, respectively), with null heterogeneity (Additional file 1: Table S1). All SGLT-2i reduced the risk of HF; among GLP-1RA, efpeglenatide only did so (Fig. 5).

### All-cause death

**Fig. 6:**
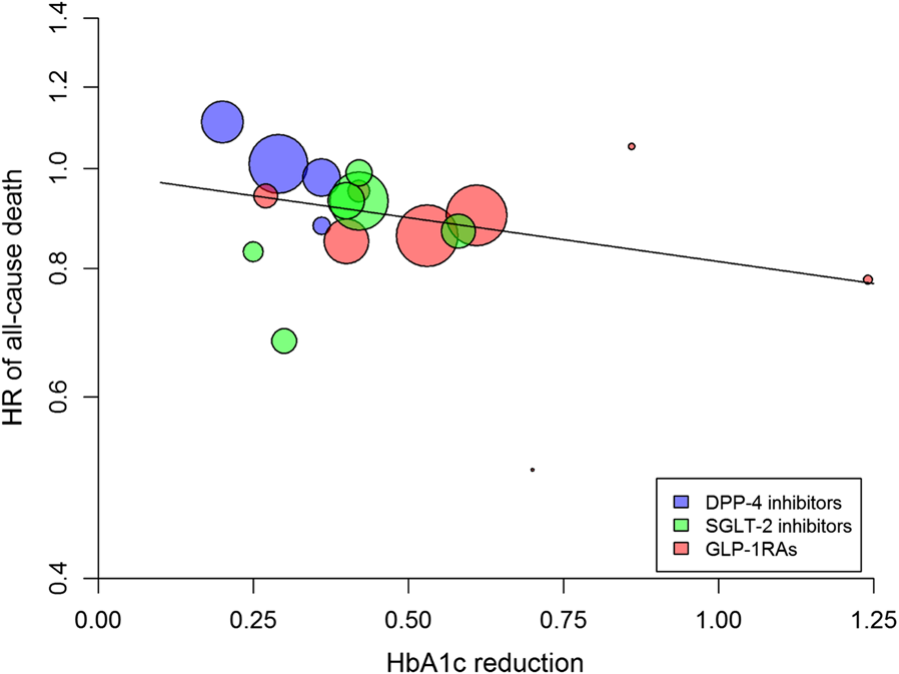
Meta-regression analysis between the differences in achieved HbA1c at the end of CVOTs and the corresponding hazard ratio (HR) for all-cause death in patients with type 2 diabetes participating in 18 CVOTs

Overall, there was a significant (P = 0.001) 9% risk reduction of all-cause death associated with the use of newer drugs compared with placebo, with significant heterogeneity (I^2^ = 55.6%, P = 0.012) (Additional file 1: Table S1) and some evidence of publication bias (Egger test, P = 0.074). However, the trim-and-fill method indicated that this publication bias did not impact the estimate. There was a nonsignificant association between the reduction in achieved HbA1c and the HR for all-cause death (beta =  − 0.196, P = 0.192, variance explained = 24%) (Fig. 6, Table 2). The use of both GLP-1RA and SGLT-2i was associated with a significant 12% and 13% risk reduction in all-cause death, respectively, with a significant heterogeneity for SGLT-2i (Additional file 1: Table S1). The trials that showed a significant benefit on all-cause death were LEADER, EXSCEL and PIONEER 6 for GLP-1RA and EMPA-REG OUTCOME for SGLT-2i.

## Discussion

This analysis used the tools of meta-analysis and meta-regression to explore the association between changes in HbA1c observed in the treatment arms of CVOTs, as compared to the placebo arms, and the reduction of MACE. The results of the meta-regression analysis of the 18 CVOTs in 161,156 patients with type 2 diabetes show that the reduction of HbA1c during treatment with DPP-4i, GLP-1RA or SGLT-2i is associated with reduction of MACE, explaining almost all (R^2^ = 97%) of the between-study variance. The risk reduction of MACE was almost completely driven by the reduction of non-fatal stroke, whose association explains 100% of between-study variance, and is unique in holding this relationship among MACE components. In the pooled analysis of the 18 CVOTs, we found a 10% reduction of MACE risk in patients treated with the newer antihyperglycemic drugs, which remained significant for GLP-1RA and SGLT-2i (14% and 11% risk reduction, respectively), and was null for DPP-4i. The inclusion of VERTIS, SCORED and AMPLITUDE-O trials in the qualitative synthesis confirm and extend results coming from our previous meta-analysis [4], corroborating the robustness of these findings.

To minimize the confounding effect of differences in glycemic control, all the CVOTs were designed to promote glycemic equipoise between the two arms (intervention versus placebo) of each trial. However, it was not mandatory that glycemic equipoise was a prerequisite for the completion of the trial; therefore, participants in the placebo group had worse glycemic control as compared with those in the treatment group. Although intensive glycemic control has an imperfect role in reducing the CV burden of patients with type 2 diabetes, it can reduce the risk of MACE by 9% [38], which is not quite dissimilar to the overall reduction of MACE (10%) found in the present meta-analysis. Moreover, a meta-regression analysis of randomized controlled trials comparing intensive blood glucose control obtained with conventional diabetes therapy (mainly insulin) with a less intensive regimen showed limited benefit of intensive glycemic control in people with type 2 diabetes and myocardial infarction, with a significant risk of serious hypoglycemia [39]. In general, the CVOTs that obtained the best glycemic equipoise between treatments presented a smallest effect on MACE. Accordingly, DPP-4i produced the least reduction of HbA1c (mean reduction = − 0.30%) and the least effect on MACE (HR, 0.99). On the other hand, the relation does not entirely apply for SGLT-2i because, at the almost same level of HbA1c reduction (mean reduction = − 0.39%), the risk of MACE was reduced by 11% (HR = 0.89), suggesting for SGLT-2i additional CV benefits independent of glucose regulation. The mean HbA1c reduction obtained in the eight CVOTs with GLP-1RA was greater (mean HbA1c reduction = − 0.63%) than that obtained with DPP-4i or SGLT-2i and was associated with a greater reduction of MACE risk.

The association between reduction in HbA1c levels by newer drugs and the risk of MACE was entirely driven by non-fatal stroke, suggesting that blood glucose reduction may play a more important role than previously thought in reducing the risk of non-fatal stroke during treatment with the newer glucose-lowering drugs. Reduced risk of hypoglycemia observed with these drugs may also have played a role, as severe hypoglycemic episodes within the previous 3 months were associated with increased risk for MACE in the Veterans Affairs Diabetes Trial [40]. With this perspective, GLP-1RA may be considered in patients with type 2 diabetes and a previous stroke or those at high risk of stroke [41]. On the other hand, the lack of the association between HbA1c reduction and the risk of other atherosclerosis-based conditions, including myocardial infarction, deserves further investigation. The higher prevalence of an established coronary heart diseases (nearly 60% in the global population included in the trials), which can predispose to a greater risk of new events, may have played a role in blunting the potential favorable effects of blood glucose reduction with newer glucose-lowering drugs on the heart.

Diabetes continues to be a relevant cause of disability worldwide, despite the availability of glucose-lowering drugs which have proved to be protective against major cardiovascular events in people at high risk of vascular complications [42, 43]. The residual cardiorenal risk after successful glycemic control with new glucose-lowering agents is similar for GLP-1RA and SGLT-2i and null for DPP-4i for MACE [44]. SGLT-2i, as compared with GLP-1RA, removed more risk for both kidney outcomes and HF, whereas DPP-4i have no clinically important benefits on these outcomes [44]. Moreover, beyond their glucose-lowering effects, these drugs have several pleiotropic protective properties, which include anti-inflammatory and immunomodulatory activities, antifibrotic and antithrombotic effects, and vascular endothelial protective properties which may be responsible for their potential favorable impact on clinical outcomes in diabetic people with COVID-19 [5, 45]. There is also evidence from observational studies that DPP-4i decrease the risk for COVID-19-related death [46, 47].

This study has limitations. Meta-regression evaluates mean data that do not necessarily correspond to individual patient data. In order to minimize the risk of ecological fallacy, our results should therefore be considered as exploratory, and thus always interpreted in conjunction with the effect from the subset of studies most relevant to the patients. This study has also strengths. The number of studies included in the meta-regression is higher than the minimum of 10 recommended by the Cochrane Collaboration Handbook [48]; moreover, all the CVOTs were powered to assess MACE outcome.

## Conclusions

In conclusion, blood glucose lowering may play a role in reducing the risk of MACE during treatment with the newer agents. However, this mediation is highly significant for non-fatal stroke only; the other two components of MACE (non-fatal MI and CV death), as well as HF and all-cause death, are not associated with improvement of glycemic control during treatment. The possible contribution of glucose lowering in mediating part of CV benefit on MACE by GLP-1RA or SGLT-2i does not influence their indications for the patient with type 2 diabetes. Current recommendations by ADA [6] state that GLP-1RA should be considered in patients with established, or at a high risk of CV disease, and SGLT-2i for patients with heart failure or chronic kidney disease. Acknowledging the potential contributive role of glucose lowering in decreasing, at least in part, MACE risk of patients with type 2 diabetes may hopefully overcome some residual reluctance of clinicians who still prefer older diabetes drugs without a clear evidence of CV protection.

## Supplementary Information

**Additional file 1**. Additional figure and table.

## Data Availability

All data generated or analyzed during this study are included in this published article and in its Additional file.

## Abbreviations

ADA: American Diabetes Association
CV: Cardiovascular
CVOTs: Cardiovascular outcome trials
GLP-1RA: Glucagon-like peptide-1 receptor agonists
HR: Hazard ratios
MACE: Major cardiovascular events
SGLT-2i: Sodium–glucose transporter-2 inhibitors
T2DM: Type 2 diabetes mellitus
