# Supplementary material for: Improvement of glycemic control and reduction of major cardiovascular events in 18 cardiovascular outcome trials: an updated meta-regression
Source: Cardiovasc Diabetol. 2021 Oct 18;20:210. doi: 10.1186/s12933-021-01401-8 (PMC8522255; doi:10.1186/s12933-021-01401-8)
Supplement: Supplementary file 1 — Additional file 1. Additional figure and table. [file 12933_2021_1401_MOESM1_ESM.docx]

Improvement of glycemic control and reduction of major cardiovascular events in 18 cardiovascular outcome trials: An updated meta-regression

**Maria Ida Maiorino, Miriam Longo, Lorenzo Scappaticcio, Giuseppe Bellastella, Paolo Chiodini, Katherine Esposito, Dario Giugliano**

**Additional files**

Additional file 1: Figure S1……………………………………………………………………………………………………… page 2

Additional file 1: Table S1 ….…………………………………………………………………………………………………. page 3

Additional file 1: Figure S2……………………………………………………………………………………………………… page 4

PRISMA CHECKLIST………………………………………………………………………………………………………………page 5

Protocol of the study…………………………………………………………………………………………………………..page 8

Extraction document…………………………………………………………………………………………………………..page 10

Records identified through Pub Med: 135

**Screening**

**Included**

**Eligibility**

Records identified through other sources (EMBASE: 48; ClinicalTrials.gov.: 27; Cochrane Database: 27; manual search: 5

**Identification**

Records after duplicates removed (n = 187)

Records excluded based on titles and abstracts (n = 169)

- Reviews, comments, editorials (n=96)
- No CVOT or not contributory secondary analysis (28)
- Design manuscript (15)
- Meta-analyses (30)

Unique trials included (n = 18)

Total records from all databases: 242

Duplicates removed
(n = 55)

**Additional file 1: Figure S1.** Study selection

**Additional file 1: Table S1. Preplanned statistical analyses**

| Outcome | Trials | Estimate | 95% CI | P-value | I^2^ (%) | P-value |
| --- | --- | --- | --- | --- | --- | --- |
|  |  | HR |  |  |  | Q-test |
| MACE |  |  |  |  |  |  |
| All | 18 | 0.90 | 0.86-0.94 | <0.001 | 45.2 | 0.040 |
| DPP-4i | 4 | 0.99 | 0.93-1.05 | 0.787 | 0.0 | 0.950 |
| GLP-1RA | 8 | 0.86 | 0.80-0.93 | <0.001 | 50.1 | 0.082 |
| SGLT-2i | 6 | 0.89 | 0.84-0.94 | <0.001 | 0.0 | 0.449 |
| CV-death |  |  |  |  |  |  |
| All | 18 | 0.88 | 0.83-0.94 | <0.001 | 44.3 | 0.047 |
| DPP-4i | 4 | 0.98 | 0.89-1.08 | 0.654 | 4.0 | 0.379 |
| GLP-1RA | 8 | 0.87 | 0.79-0.95 | 0.002 | 19.3 | 0.330 |
| SGLT-2i | 6 | 0.84 | 0.74-0.96 | 0.011 | 58.4 | 0.043 |
| Non-fatal MI |  |  |  |  |  |  |
| All | 18 | 0.92 | 0.87-0.98 | 0.007 | 36.5 | 0.108 |
| DPP-4i | 4 | 1.00 | 0.92-1.10 | 0.928 | 0.0 | 0.445 |
| GLP-1RA | 8 | 0.91 | 0.83-1.00 | 0.039 | 34.1 | 0.173 |
| SGLT-2i | 6 | 0.87 | 0.79-0.96 | 0.008 | 29.8 | 0.238 |
| Non-fatal stroke |  |  |  |  |  |  |
| All | 18 | 0.91 | 0.84-0.98 | 0.012 | 21.3 | 0.206 |
| DPP-4i | 4 | 1.00 | 0.87-1.14 | 0.949 | 0.0 | 0.664 |
| GLP-1RA | 8 | 0.84 | 0.76-0.93 | 0.001 | 0.0 | 0.589 |
| SGLT-2i | 6 | 0.93 | 0.79-1-10 | 0.388 | 53.9 | 0.086 |
| Hospitalization for HF |  |  |  |  |  |  |
| All | 18 | 0.84 | 0.77-0.93 | <0.001 | 69.2 | <0.001 |
| DPP-4i | 4 | 1.05 | 0.91-1.22 | 0.499 | 53.9 | 0.058 |
| GLP-1RA | 8 | 0.90 | 0.83-0.98 | 0.016 | 0.0 | 0.673 |
| SGLT-2i | 6 | 0.68 | 0.62-0.75 | <0.001 | 0.0 | 0.923 |
| All-cause death |  |  |  |  |  |  |
| All | 18 | 0.91 | 0.85-0.96 | 0.001 | 55.6 | 0.012 |
| DPP-4i | 4 | 1.01 | 0.93-1.10 | 0.803 | 16.9 | 0.322 |
| GLP-1RA | 8 | 0.88 | 0.82-0.95 | 0.001 | 26.1 | 0.349 |
| SGLT-2i | 6 | 0.87 | 0.79-0.97 | 0.009 | 59.3 | 0.049 |

MACE, major cardiovascular events; MI, myocardial infarction; HF, heart failure.


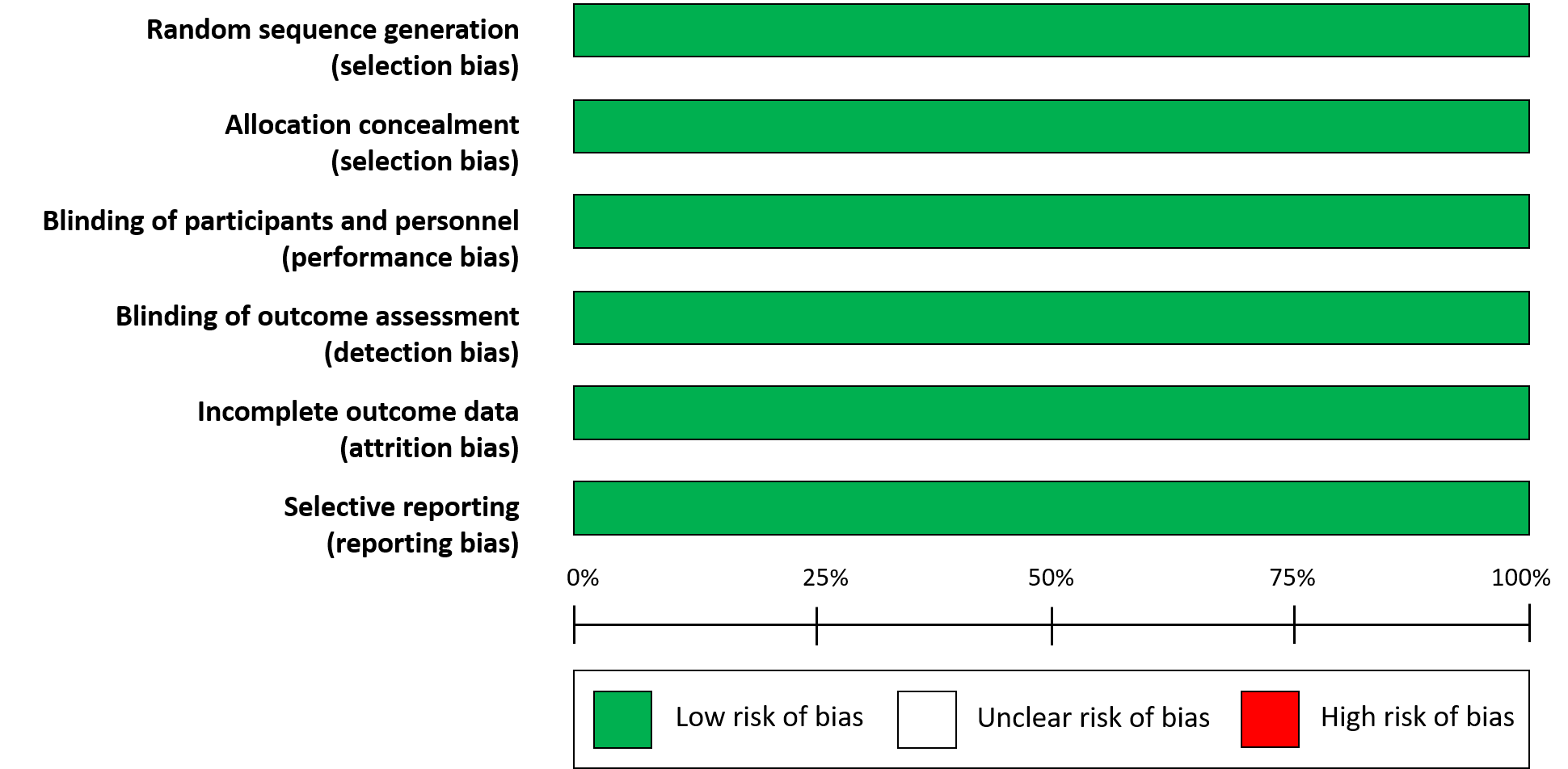


**Additional file 1: Figure S2**. Cochrane risk of bias (graph) for the 18 CVOT trials

| **Section/topic** | | | **#** | **Checklist item** | **Reported on page #** |  |
| --- | --- | --- | --- | --- | --- | --- |
| **TITLE** | | | | |  |  |
| Title | | | 1 | Identify the report as a systematic review, meta-analysis, or both. | 1 |  |
| **ABSTRACT** | | | | |  |  |
| Structured summary | | | 2 | Provide a structured summary including, as applicable: background; objectives; data sources; study eligibility criteria, participants, and interventions; study appraisal and synthesis methods; results; limitations; conclusions and implications of key findings; systematic review registration number. | 2 |  |
| **INTRODUCTION** | | | | |  |  |
| Rationale | | | 3 | Describe the rationale for the review in the context of what is already known. | 4 |  |
| Objectives | | | 4 | Provide an explicit statement of questions being addressed with reference to participants, interventions, comparisons, outcomes, and study design (PICOS). | 4-5 |  |
| **METHODS** | | | | |  |  |
| Protocol and registration | 5 | | | Indicate if a review protocol exists, if and where it can be accessed (e.g., Web address), and, if available, provide registration information including registration number. | 5 |  |
| Eligibility criteria | 6 | | | Specify study characteristics (e.g., PICOS, length of follow-up) and report characteristics (e.g., years considered, language, publication status) used as criteria for eligibility, giving rationale. | 5-6 |  |
| Information sources | 7 | | | Describe all information sources (e.g., databases with dates of coverage, contact with study authors to identify additional studies) in the search and date last searched. | 5-6 |  |
| Search | 8 | | | Present full electronic search strategy for at least one database, including any limits used, such that it could be repeated. | 5-6 |  |
| Study selection | 9 | | | State the process for selecting studies (i.e., screening, eligibility, included in systematic review, and, if applicable, included in the meta-analysis). | 6 |  |
| Data collection process | 10 | | | Describe method of data extraction from reports (e.g., piloted forms, independently, in duplicate) and any processes for obtaining and confirming data from investigators. | 6 |  |
| Data items | 11 | | | List and define all variables for which data were sought (e.g., PICOS, funding sources) and any assumptions and simplifications made. | 6 |  |
| Risk of bias in individual studies | 12 | | | Describe methods used for assessing risk of bias of individual studies (including specification of whether this was done at the study or outcome level), and how this information is to be used in any data synthesis. | 6-7 |  |
| Summary measures | 13 | | | State the principal summary measures (e.g., risk ratio, difference in means). | 7 |  |
| Synthesis of results | 14 | | | Describe the methods of handling data and combining results of studies, if done, including measures of consistency (e.g., I^2^) for each meta-analysis. | 7 |  |
| Risk of bias across studies | | 15 | | | Specify any assessment of risk of bias that may affect the cumulative evidence (e.g., publication bias, selective reporting within studies). | 7 |
| Additional analyses | | 16 | | | Describe methods of additional analyses (e.g., sensitivity or subgroup analyses, meta-regression), if done, indicating which were pre-specified. | 7 |
| **RESULTS** | | | | | |  |
| Study selection | | 17 | | | Give numbers of studies screened, assessed for eligibility, and included in the review, with reasons for exclusions at each stage, ideally with a flow diagram. | 7-10 |
| Study characteristics | | 18 | | | For each study, present characteristics for which data were extracted (e.g., study size, PICOS, follow-up period) and provide the citations. | 7-10 |
| Risk of bias within studies | | 19 | | | Present data on risk of bias of each study and, if available, any outcome level assessment (see item 12). | 7-10 |
| Results of individual studies | | 20 | | | For all outcomes considered (benefits or harms), present, for each study: (a) simple summary data for each intervention group (b) effect estimates and confidence intervals, ideally with a forest plot. | 7-10 |
| Synthesis of results | | 21 | | | Present results of each meta-analysis done, including confidence intervals and measures of consistency. | 7-10 |
| Risk of bias across studies | | 22 | | | Present results of any assessment of risk of bias across studies (see Item 15). | 7-10 |
| Additional analysis | | 23 | | | Give results of additional analyses, if done (e.g., sensitivity or subgroup analyses, meta-regression [see Item 16]). | 7-10 |
| **DISCUSSION** | | | | | |  |
| Summary of evidence | | | 24 | | Summarize the main findings including the strength of evidence for each main outcome; consider their relevance to key groups (e.g., healthcare providers, users, and policy makers). | 11-13 |
| Limitations | | | 25 | | Discuss limitations at study and outcome level (e.g., risk of bias), and at review-level (e.g., incomplete retrieval of identified research, reporting bias). | 11-13 |
| Conclusions | | | 26 | | Provide a general interpretation of the results in the context of other evidence, and implications for future research. | 11-13 |
| **FUNDING** | | | | | |  |
| Funding | | | 27 | | Describe sources of funding for the systematic review and other support (e.g., supply of data); role of funders for the systematic review. | 13 |

**Protocol for the association between reduction of major cardiovascular events (MACE) and improvement of glycemic control in 18 cardiovascular outcome trials (CVOTs): a meta-analysis and meta-regression.**

- Broad question 1: What is the effect of newer drugs (DPP-4 inhibitors, GLP-1 receptor agonists, SGLT-2 inhibitors) on the risk of MACE in patients with type 2 diabetes?
- Broad question 2: Is there any association between the reduction of major cardiovascular events (MACE) and improvement of glycemic control, as assessed by the reduction of HbA1c at the end of the trial?
- Specific question 1: What is the effect of newer drugs (DPP-4 inhibitors, GLP-1 receptor agonists, SGLT-2 inhibitors) on the risk of MACE components (non-fatal myocardial infarction, cardiovascular death, non-fatal stroke) in patients with type 2 diabetes?
- Specific question 2: Is there any association between improvement of glycemic control during treatment and each MACE component, as well as hospitalization for heart failure or all-cause death?

The answer to these specific points was sought by evaluating RCTs performed in adults with type 2 diabetes, comparing add-on therapy with any DPP-4 inhibitor, GLP-1 receptor agonist or SGLT-2 inhibitor with placebo, and including in the outcome (either primary or secondary) MACE and its components (cardiovascular death, non-fatal myocardial infarction, non-fatal stroke), as well as other outcomes required by regulatory agencies for cardiovascular safety studies in diabetes. The primary efficacy outcomes for this meta-analysis were 1) the effect of DPP-4 inhibitors, GLP-1 receptor agonists, and SGLT-2 inhibitors on the risk of MACE compared with placebo and 2) the relation between improvement of glycemic control (ΔHbA1c –reduction of HbA1c as compared with placebo) and reduction of MACE. Additional analyses were conducted on MACE components, all-cause death, and hospitalization for heart failure.

*The review followed the outlines of PICO (study characteristics):*

1. Population: The population to be included in the review consisted of subjects with type 2 diabetes at baseline.
2. Exposure: Any DPP-4 inhibitor, GLP-1 receptor agonist, SGLT-2 inhibitor compared with placebo.
3. Comparisons: Subjects with type 2 diabetes.
4. Outcomes: Risk of MACE, its separate components (CV death, non-fatal myocardial infarction, non-fatal stroke), hospitalization for heart failure, all-cause death; relation between improvement of glycemic control (ΔHbA1c –reduction of HbA1c as compared with placebo) and reduction of MACE or other cardiovascular outcomes.

Published articles were considered eligible for this review if they: were RCTs evaluating adult with type 2 diabetes, comparing add-on therapy with any DPP-4 inhibitor, GLP-1 receptor agonist or SGLT-2 inhibitor with placebo, included in the outcome (either primary or secondary) MACE, its components (cardiovascular death, non-fatal myocardial infarction, or non-fatal stroke), as well as other outcomes required by regulatory agencies for cardiovascular safety studies in diabetes.

| **Extraction document** | | | |  |  |  |  |  |  |
| --- | --- | --- | --- | --- | --- | --- | --- | --- | --- |
| Trial/year of | Study drug/ | Participants | Mean age | Baseline | Δ A1C (%) | Type of prior CV | Principal | Secondary | Study |
| publication | Mean follow up | (n) | (years) | A1C (%) |  | disease | outcome | outcome | funder |

| **Extraction document (continued)** | | | |  |  |  |  |  |  |
| --- | --- | --- | --- | --- | --- | --- | --- | --- | --- |
| \| MACE (HR \| Nonfatal MI \| CV death \| Nonfatal stroke \| Heart failure \| \| --- \| --- \| --- \| --- \| --- \| \| (and 95% CI) \| (HR and CI) \| (HR and CI) \| (HR and CI) \| (HR and CI) \| |  |  |  |  |  |  |  |  |  |
|  |  |  |  |  |  |  |  |  |  |

| **Extraction document (continued)** | | | |  |  |  |  |  |  |
| --- | --- | --- | --- | --- | --- | --- | --- | --- | --- |
| \| All-cause mortality \| \| --- \| \| (HR and 95% CI) \| |  |  |  |  |  |  |  |  |  |
